# The role of urban municipal governments in reducing health inequities: A meta-narrative mapping analysis

**DOI:** 10.1186/1475-9276-9-13

**Published:** 2010-05-25

**Authors:** Patricia A Collins, Michael V Hayes

**Affiliations:** 1 Department of Health, Aging & Society, McMaster University, Hamilton, Ontario, Canada; 2 Faculty of Health Sciences, Simon Fraser University, Burnaby, British Columbia, Canada

## Abstract

**Background:**

The 1986 Ottawa Charter for Health Promotion coincided with a preponderance of research, worldwide, on the social determinants of health and health inequities. Despite the establishment of a 'health inequities knowledge base', the precise roles for municipal governments in reducing health inequities at the local level remain poorly defined. The objective of this study was to monitor thematic trends in this knowledge base over time, and to track scholarly prescriptions for municipal government intervention on local health inequities.

**Methods:**

Using meta-narrative mapping, four bodies of scholarly literature - 'health promotion', 'Healthy Cities', 'population health' and 'urban health' - that have made substantial contributions to the health inequities knowledge base were analyzed over the 1986-2006 timeframe. Article abstracts were retrieved from the four literature bodies using three electronic databases (PubMed, Sociological Abstracts, Web of Science), and coded for bibliographic characteristics, article themes and determinants of health profiles, and prescriptions for municipal government interventions on health inequities.

**Results:**

1004 journal abstracts pertaining to health inequities were analyzed. The overall quantity of abstracts increased considerably over the 20 year timeframe, and emerged primarily from the 'health promotion' and 'population health' literatures. 'Healthy lifestyles' and 'healthcare' were the most commonly emphasized themes in the abstracts. Only 17% of the abstracts articulated prescriptions for municipal government interventions on local health inequities. Such interventions included public health campaigns, partnering with other governments and non-governmental organizations for health interventions, and delivering effectively on existing responsibilities to improve health outcomes and reduce inequities. Abstracts originating from Europe, and from the 'Healthy Cities' and 'urban health' literatures, were most vocal regarding potential avenues for municipal government involvement on health inequities.

**Conclusions:**

This study has demonstrated a pervasiveness of 'behavioural' and 'biomedical' perspectives, and a lack of consideration afforded to the roles and responsibilities of municipal governments, among the health inequities scholarly community. Thus, despite considerable research activity over the past two decades, the 'health inequities knowledge base' inadequately reflects the complex aetiology of, and solutions to, population health inequities.

## Background

### Connections and divergences between urban planning and public health

There is a long-standing connection between the manner in which cities are planned and managed, and the health outcomes that manifest among urban dwellers. The 19^th ^century sanitation movement of the Victorian era, for instance, promoted a union of public health and urban planning that spurned an array of sanitary-based engineering interventions, including sewerage and waste management systems, development of potable drinking water, and public health inspection [1-3]. Along with broader social, economic and political changes (e.g., establishment of social safety nets, public education and healthcare systems, transitions to service- and knowledge-based economies, suffrage, civil rights' movement), the institutionalization and perpetuation of these sanitary-based interventions by municipal governments over the 20^th ^century made substantial contributions to improvements in longevity in the developed world [4,5].

With the establishment of germ theory in the late 1800s, however, the common ground shared by public health and urban planning did not persist [6]. Urban planning in North America rigidly applied a Haussmann-inspired approach to zoning that created cities with functionally and economically homogeneous neighbourhood units [7], generating a legacy of geographically disconnected urban agglomerations negotiable only through widespread use of the automobile [8]. Public health turned to laboratory medicine and immunization-based interventions [3], which have been critiqued for bearing limited influence on improvements in longevity [9].

### Emergence of health inequities research

In the 1970s and early 80s, seminal documents were published linking population health outcomes with non-medical factors [5,9-11]. Since this time, we have witnessed a preponderance of research documenting patterns, determinants of, and strategies to reduce, health inequities at the population level, where *health inequities *refer to health differences attributable to disparities in advantages, opportunities, or exposures in social, economic, political, cultural, environmental and/or some other dimensions. The 'health inequities knowledge base' that has flourished since the 1970s has emerged from two distinct, but related, strands of research that, in this paper, will be referred to as 'health promotion' and 'population health' [12].

While health promotion *practice *originates in the work of health educators [13], the inception of 'health promotion' as a line of academic inquiry can be dated to 1986, when it was defined in the first issue of the *American Journal of Health Promotion *as "the art and science of helping people change their lifestyle to move toward a state of optimal health" [14]. Recognizing the limitations of this lifestyle-oriented definition, health promotion researchers strode quickly to broaden the scope of health promotion to advocate for environmental and societal changes that would reduce population health inequities [13]. As such, the Ottawa Charter for Health Promotion, released during the first international conference of its kind in 1986, described the principles of health promotion as social justice, equity, peace, and sustainability, and recommended interventions that facilitated community empowerment and capacity building, and bottom-up approaches to defining problems and developing solutions [15-18].

The environmental direction of the field of health promotion gave rise to the Healthy Cities movement [13] (or the Healthy Communities Project as it was in Canada [19]). Initiated by the WHO [20], the Healthy Cities movement applied notions of health-related empowerment to communities and cities [16-18], by facilitating community participation to address local health problems [18,21]. Early recommendations for relevant stakeholders in the Healthy Cities movement were to develop inter-sectoral partnerships, engage community partners, develop indicators of success, and focus on preventive programs [19,22-24]. More recent Healthy Cities initiatives have focused on promoting safety (e.g., ensuring housing quality, injury prevention, crime reduction), environmental quality (e.g., reducing water and air pollution), and physical activity [25,26].

The second strand of research, 'population health', originates from early observations made by social epidemiologists on the socially graded nature of population health outcomes [27,28]. As with social epidemiology, population health researchers are similarly concerned with "investigating social determinants of population distributions of health, disease, and wellbeing" [29], p.693], but they tend *also to*, or sometimes *only*, examine policies and interventions that shape or target the social determinants of population health inequities [30,31]. As such, researchers from an array of disciplinary backgrounds (i.e., social epidemiology as well as health policy, health economics, sociology, geography, etc.) have made key contributions to population health. The Population Health Approach, as it is known in Canada and internationally [32,33], draws on the social determinants of health (SDOH) - a conceptual framework derived from social epidemiology for describing the multitude of factors (and potential policy levers) that mediate social gradients in population health outcomes [34] - to advocate primarily for top-down policy interventions to tackle health inequities [35]. (Recognizing complementarities in ideals and objectives, it is noteworthy that efforts have been made in recent years, led by Canadian researchers, to reconcile epistemological divides [36,37] to advance a new field of Population Health Promotion [38-41].)

The field of urban health emerged in the 1990s in response to global patterns of urbanization, the growing burden of disease among vulnerable populations, and pervasive socioeconomic inequities within urban systems [42]. Urban health draws from social epidemiology through its "explicit investigation of the relation between the urban context and *population distribution of health and disease*" [emphasis added] [43], but it differs from social epidemiology by adopting and applying a range of theoretical perspectives and methodological approaches to examine the questions posed [44]. Urban health also parallels the multi-disciplinarity of population health research through contributions from a range of academic fields, including anthropology, health services research, health geography, urban sociology, environmental science, and others [45].

Thus, considerable scholarly attention, from various disciplinary perspectives, has been paid to population health inequities in the latter part of the 20^th ^century, and has generated a rich knowledge base from which to draw solutions for change. Yet, reductions in health inequities in North America were considerably greater in the first half of the 20th century; for example, disparities in life expectancy between white and black US men dropped from 14 years in 1900 to 8 years in 1950 to 7 years in 2000 [46]. Indeed, recent research has demonstrated the persistence of social gradients in health among the Canadian population as the 21st century has arrived [47]. Thus, while exposure to abject living conditions (and socioeconomic inequities therein) in the 19^th ^century may have stimulated the sanitation era, and its accompanying union of public health and urban planning, it would appear that the health inequities knowledge base of today has not inspired a comparable movement to alleviate socioeconomic inequities in population health.

### Importance of the Municipal Level in Addressing Health Inequities

Internationally, population growth is occurring predominantly in urban agglomerations [48]. In Canada, for instance, 45% of the country's population is living in one of six large metropolitan regions [49]. Because these urban systems act as socio-spatial sorting mechanisms [50], social gradients in health are manifested and perpetuated across socio-economically homogenous neighbourhood units [51-53]. These health inequities are not limited to urban core areas, as evidence is mounting of the detrimental impacts of sprawling development on population health outcomes (e.g., high rates of obesity, mental illness, and respiratory problems) [54-61]. Thus, social gradients in health can be created and exacerbated when municipal governments (or comparable governmental bodies operating locally) are unable to plan, deliver, and manage equitable and viable spaces to live amidst rapid population growth [62]. Given these trends in urban growth and land-use patterns, and that much policy activity on population health happens outside of health ministries and departments (by virtue of the complexity and multidisciplinary nature of the social determinants of health [63]), it appears that policies and plans implemented by municipal governments - even those without health mandates - are important components of the larger project of addressing population health inequities.

The capacities of municipal governments to take action on population health inequities at the local level are highly context-dependent, and contingent on the form, function, jurisdictional powers and priorities of these governments - all of which vary internationally, nationally, and in some cases, regionally. The establishment of mayoral representation, for instance, varies considerably between countries, from direct election (e.g., most American nations, Italy, Poland, Japan, New Zealand, Russia, South Korea), to indirect election through council appointments (e.g., Denmark, Portugal, France, India, Vietnam), to appointments by central governments (e.g., Belgium, Luxembourg, Netherlands, China, Laos, Malaysia) [64-66]. Geographical jurisdictions of municipal governments range from urban agglomerations, cities or regions, to towns, boroughs, villages, districts, counties, and communes [64-67]. The scope of municipal governments' responsibilities and priorities vary depending on the size and sophistication of these institutions (e.g., range of departments), constitutional authority, availability of resources (from senior governments, taxpayers, etc.), and the types of issues warranting government intervention (e.g., basic infrastructure requirements in municipalities in developing nations versus provision of education and welfare services in some developed nations' cities) [64-66].

Despite variations in what they are and how they operate within their jurisdictions, municipal governments, and municipalities more generally, possess features that position them (to varying degrees) to address population health inequities. Across international, national, and regional jurisdictions, municipal responsibilities for a number of different sectors are commonly held, including culture & leisure, education, environment, health & social services, housing, planning, public safety, transportation, water, and/or waste [64-66,68]. Municipal governance models are increasingly shifting from managerialism (i.e., delivering on slated responsibilities) to entrepreneurialism (i.e., protection and promotion of local economies through the development of new enterprises) [69], and increasingly involve stakeholders beyond municipal governments, such as private businesses, non-profit organizations, and local residents (e.g., urban regimes in the USA [70], government-coordinated public-private partnerships in Europe [71]). Additionally, municipalities (especially urban agglomerations and cities) are sites of major hospitals, universities, think tanks, influential non-governmental organizations, a well-organized public health sector, and organized interest groups with powerful communication skills and significant capacity to mobilize [72]. Thus, municipalities offer promising sites for interventions on population health inequities because of the combined opportunities for top-down policy interventions delivered by municipal governments, and bottom-up participation from potentially engaged, mobilized, and knowledgeable local stakeholders.

### Study Objectives

Because of these characteristics, many urban health scholars believe municipal governments are a fundamental component of initiatives to reduce population health inequities, triggering calls for a reinvigoration of the spirit of the 19^th ^century union of public health and urban planning by assigning greater responsibility and authority to municipal governments to tackle population health inequities [1,3,4,62,73-75]. A number of researchers have documented a tremendous gap between knowledge and policy action to tackle social gradients in health [35,41,76-82]. Yet, the roles and capacities of urban municipalities to address population health inequities, as perceived by both researchers and urban municipal policy-makers themselves, have been particularly neglected areas of study. While the Healthy Cities movement has been active in prescribing avenues for municipal activity (primarily in non-academic/grey literature [25,26,83-85]), it remains to be empirically demonstrated how other health inequities literatures have implicated municipalities, the precise nature of these implications, and the manner in which these implications are taken up by relevant municipal actors and institutions.

The purpose of this study was to address the first two of these deficiencies in the research, by monitoring thematic trends in the health inequities knowledge base over time, and to track scholarly prescriptions for municipal government intervention on local health inequities. While a Canadian and urban lens has been applied to this study, the findings from this analysis of an international knowledge base will foster a greater understanding of the challenges and issues associated with translating the health inequities research into policy action within a variety of municipal and geographical contexts.

## Methods

### Methodology

Meta-narrative mapping - the process of "plotting how a particular research tradition has unfolded over time and placing this dynamic tradition within a broader field of enquiry" [86], p.349] - was employed to determine when and how municipal governments have been implicated in the scholarly literature for reducing local health inequities. This novel methodological approach combines the analytical dimensions of traditional narrative research - storytelling, historicity, context, and human relations - with the comprehensiveness and rigor pursued in systematic literature reviews [86-88]. Among others, meta-narrative mapping has been identified as a useful methodological technique in the synthesis of vast and complex evidence bases to inform policy-making processes [89]. While this methodological approach offers utility in capturing the essential features of the health inequities knowledge base over time, the breadth of this analysis also compels readers to be cautious and critical in drawing inferences on municipal-level interventions for health inequities, as the patterns in, causes of, and local solutions to health inequities are highly contextually sensitive. For instance, a recommendation for municipal governments to increase investments in inner-city parks and recreation facilities by a Canadian scholar may have limited relevance to municipalities operating in developing countries that lack basic municipal infrastructure like roads, sewage, or water, or even to other Canadian municipalities that may not experience substantial geographic disparities in the quality of parks and recreation facilities.

### Parameters and Strategy for Literature Search

**Figure 1:**
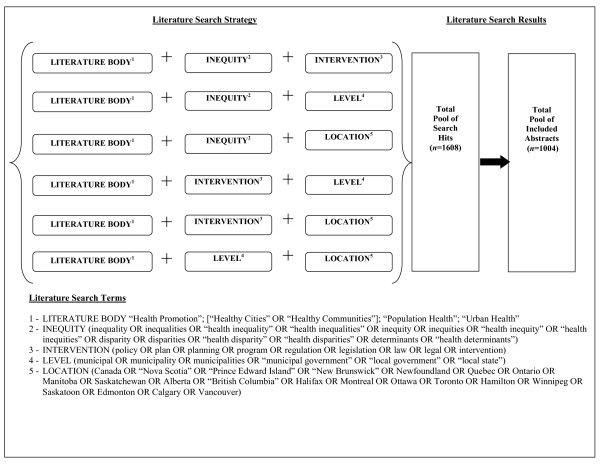
**Search Strategy for Health Inequities Abstracts**.

Four bodies of literature on health inequities - 'health promotion' (HP), 'Healthy Cities' (HC), 'population health' (PH), and 'urban health' (UH) - were examined for the meta-narrative mapping analysis. These four literature bodies were chosen because, as discussed earlier, they have made the most significant scholarly contributions to understanding *patterns of *health inequities, and identifying and describing *interventions to reduce *health inequities. While literature from other fields, such as social epidemiology, political science, health geography, sociology, or medical anthropology, have made important contributions to the study of population health inequities and related interventions, these contributions have been made on a more *ad hoc *basis than those made by the bodies of literature that were included in this study. It is possible that, by limiting the searches to these four bodies of literature, our findings may under-represent the true scope of literature pertaining to population health inequities (e.g., overlooking articles from the policy sciences on the determinants of population well-being or welfare). However, because of the breadth of the databases selected and the search terms employed (Figure 1), we are confident that relevant contributions made by researchers within disciplines not explicitly sampled here would have emerged in the searches.

Three electronic databases were selected for the literature search: PubMed, which caters to life sciences, and includes most health sciences journals; Sociological Abstracts which caters to sociology, social science, and policy science, and includes health policy, social policy, and health geography journals; and Web of Science, catering to science, social science, and arts. Web of Science was the most comprehensive of the three databases, capturing articles not found in PubMed or Sociological Abstracts. Article abstract searches were first performed in PubMed, followed by Sociological Abstracts and Web of Science, and were guided by the search strategy outlined in Figure 1. English language abstracts only were eligible for inclusion.

To capture two full decades of publication activity, the timeframe for the search was 1986 to 2006 inclusive. The year 2006 also marks the 20-year anniversary of two publications that were seminal to the establishment of health inequities research in Canada (and in some other nations) - the Ottawa Charter for Health Promotion [15] and the Epp Report [90]. Four search themes, and numerous relevant search terms, were generated to facilitate as comprehensive a search strategy as possible (Figure 1): population health inequities (INEQUITY); government-based interventions to address health inequities (INTERVENTION); interventions from municipal governments on issues related to health and well-being (LEVEL); and Canadian locations for research and/or interventions on health inequities (LOCATION). These search themes were only used in the data collection phase of the research, and were not used in the analysis of the abstracts' contents.

### Abstract Inclusion/Exclusion Criteria

Abstracts had to mention, in some capacity, differences in health outcomes or well-being, and/or the SDOH. Abstracts that discussed policy implications were also of distinct interest for review, but this was not an explicit inclusion criterion. Abstracts that described health differences in a strictly clinical scope were excluded, as were abstracts that referred to inequalities or disparities in a different context (e.g., measurement disparities). Highly technical pieces that discussed new clinical technologies, or issues related to healthcare systems and/or delivery, were excluded. Abstracts were also excluded if they contained the words "National Population Health Survey" or "Ottawa Charter for Health Promotion", but lacked any other information relevant to the review.

### Development of Abstract Codebook

A codebook was used to simplify and standardize the process of reviewing and synthesizing data. It facilitated the review of a large quantity of qualitative data, application of the same analytical standards to each case within the dataset, categorization of corresponding information, and conversion of the information reviewed into a quantitative dataset [91]. Most of the variables were developed iteratively; individual codes were first created and assigned to abstracts as string variables through a process of immersion and crystallization with the data. Once saturation of themes was reached, the list of string variables was analyzed, condensed, and converted into a list of numerical codes representing distinct entities or themes. The final abstract codebook contained three variable categories [see Additional file 1]: bibliographic characteristics; abstract content variables; and prescriptions for municipal governments.

Bibliographic characteristics of interest were body of literature (i.e., HP, HC, PH, UH) from which the abstract was retrieved; journal name; publication year; geographical region of focus (or origin); type of study described in the abstract; and population investigated by the study or target audience. Abstract contents were captured using two variables: article themes and SDOH profile. Article theme codes were developed through an inductive process of immersion with the article abstracts and saturation of article themes; codes were based not on any one particular keyword or phrase in the abstracts, but on the content area as conveyed by the abstract as a whole. Once each abstract was coded, the complete list of inductively derived article theme codes was reviewed for redundancy, and pared down to a list of 20 distinct article themes. Using Health Canada's list of twelve health determinants [92], SDOH profiles of the abstracts were captured by coding up to three different determinants (primary, secondary and tertiary determinants). Determinants coded as 'primary' were those that were given the greatest overall emphasis in the article or, in the case of equal emphasis across multiple determinants, were mentioned first; secondary and tertiary determinants were then coded based on subsequent levels of emphasis and/or timing of appearance in the abstract.

To ensure that the codebook captured the full scope of municipal government prescriptions from the abstracts, several methodological steps were taken. First, abstracts were coded 'yes' for a municipal role if they explicitly prescribed roles for municipal governments in addressing health differences at the local level and/or in improving local health outcomes. Then, the contents of these references to municipal governments were documented using string variables. Once data entry was complete, each string variable was then converted, one at a time, into a numerical code to facilitate quantification. As subsequent string variables were reviewed, and recurrent or overlapping themes emerged, existing codes were assigned and revised to reflect the expanding breadth of the code. After each abstract implicating municipal governments was assigned a code for the 'prescription', the complete list of numerical codes was reviewed for further overlaps and redundancies, synthesized, and pared down to a list of seven coherent and distinct categories of municipal government roles.

### Management and Analysis of Search Results

A total of 72 searches were performed (3 databases × 4 bodies of literature × 6 search theme combinations), generating over 1600 abstract hits for review. Every individual abstract was reviewed for relevance based on the inclusion/exclusion criteria, and then screened for redundancies. Relevant abstracts appearing in more than one electronic database were included in the total sample only once, because documenting differences in the electronic indexing system for these abstracts was not an objective of this study. Meanwhile, relevant abstracts appearing in more than one literature body were included in the sample for every literature body from which they were generated (to a maximum of four potential database entries, as four bodies of literature were examined), as documenting systematic differences in abstracts' contents between bodies of literature over time *was *an explicit objective of this study.

To facilitate review of the over 1600 hits, only abstracts, not full-text articles, were assessed. While this approach facilitated only a general analysis of the health inequities knowledge base over time (i.e., the essence of meta-narrative mapping), reviewing abstracts was empirically valid because abstracts emphasize the most important themes, findings, and actors from the full articles upon which they are based. Indeed, for time-strapped policy-makers and service providers, abstracts are often the only segments of academic articles that garner any attention. Included abstracts were assigned a numerical identifier, coded using the abstract codebook, and inputted into an SPSS^® ^database (version 15.0). Quantitative analyses of the abstracts consisted of collecting basic frequency data and performing cross-tabulations between variables.

## Results

### Number of Abstracts

A total of 1608 abstract hits were generated across the four literature bodies (HP = 972, HC = 51, PH = 555, UH = 30), and 1004 abstracts were eligible for inclusion (HP = 641, HC = 38, PH = 307, UH = 18), for an overall inclusion rate (IR) of 62.4%. Of the 1004 included abstracts, 103 of these appeared in more than one literature body (*n *= 50 were found in two and *n *= 1 was found in three bodies of literature), and thus, the contents of which were counted twice (or three times for one of the abstracts). Substantial differences in quantity of abstracts were observed between the four literature bodies, highlighting the differences in age, scope, and relative influences of these literatures on the health inequities knowledge base. Inclusion rates ranged from 55.3% for PH to 74.5% for HC, suggesting abstracts from the HC literature bore the greatest relevance to the themes of interest in this study.

### Bibliographic Characteristics

Over 40% of the abstracts were produced in and/or profiled a Canadian region, reflecting the search themes that prioritized Canadian content (Table 1). One-fifth (20.1%) of the abstracts had an American focus, while very few (2%) featured Mexico, Central and/or South America. Canadian-focused abstracts were more prevalent among HP (35.4%) and PH (56.0%) abstracts, while European-focused abstracts were more prominent in the HC (23.7%) and UH (27.8%) abstracts.

**Table 1:** Bibliographic Characteristics by Body of Literature

	Bibliographic Characteristic	Health Promotion *n *(%)		Healthy Cities *n *(%)		Population Health *n *(%)		Urban Health *n *(%)	Total *n *(%)
Geographical Region	Global, Transcontinental	83 (12.9)		9 (23.7)		60 (19.5)		2 (11.1)	154 (15.3)
	Canada	227 (35.4)		8 (21.1)		172 (56.0)		2 (11.1)	409 (40.7)
	Europe	95 (14.8)		9 (23.7)		12 (3.9)		5 (27.8)	121 (12.1)
	Australia, New Zealand, Oceania	44 (6.9)		0		14 (4.6)		0	58 (5.8)
	Asia, Africa & Middle East	28 (4.4)		3 (7.9)		5 (1.6)		4 (22.2)	40 (4.0)
	Mexico, Central & South America	9 (1.4)		2 (5.3)		7 (2.3)		2 (11.1)	20 (2.0)
	United States	155 (24.2)		7 (18.4)		37 (12.1)		3 (16.7)	202 (20.1)

Study Type	Population-Based Survey	123 (19.2)		2 (5.3)		130 (42.3)		4 (22.2)	259 (25.8)
	Experimental Study	58 (9.0)		0		21 (6.8)		0	79 (7.9)
	Program Evaluation	121 (18.9)		11 (28.9)		23 (7.5)		4 (22.2)	159 (15.8)
	Case or Qualitative Study	107 (16.7)		8 (21.1)		22 (7.2)		4 (22.2)	141 (14.0)
	Systematic or Conceptual Review	126 (19.7)		9 (23.7)		68 (22.1)		2 (11.1)	205 (20.4)
	Commentary	106 (16.5)		8 (21.1)		43 (14.0)		4 (22.2)	161 (16.0)

	Total	641 (100)		38 (100)		307 (100)		18 (100)	1004 (100)

Over one-quarter (25.8%) of the abstracts described population-based surveys and 20.4% were reviews, highlighting a lack of evaluative studies on health inequities and related interventions. Reflecting the field's strong epidemiological roots, population-based surveys constituted almost half of the PH abstracts (42.3%), while the other three bodies of literature offered more balance in terms of study type. Commonly employed study populations or target audiences were adults (20.5%), practitioners (17.6%), and researchers (15.0%). The high proportion of 'adults' as study populations is likely attributable to the large proportion of studies employing surveys, while the large number of reviews may account for the high proportion of 'practitioners' and 'researchers' as target audiences. By literature body, the most commonly employed study populations were practitioners in HP abstracts (21.7%), government in HC abstracts (31.6%), and adults in PH (32.6%) and UH (44.4%) abstracts. The focus on government suggests that the HC literature may be the most active in prescribing roles for municipal governments.

### Abstracts' Contents

**Figure 2:**
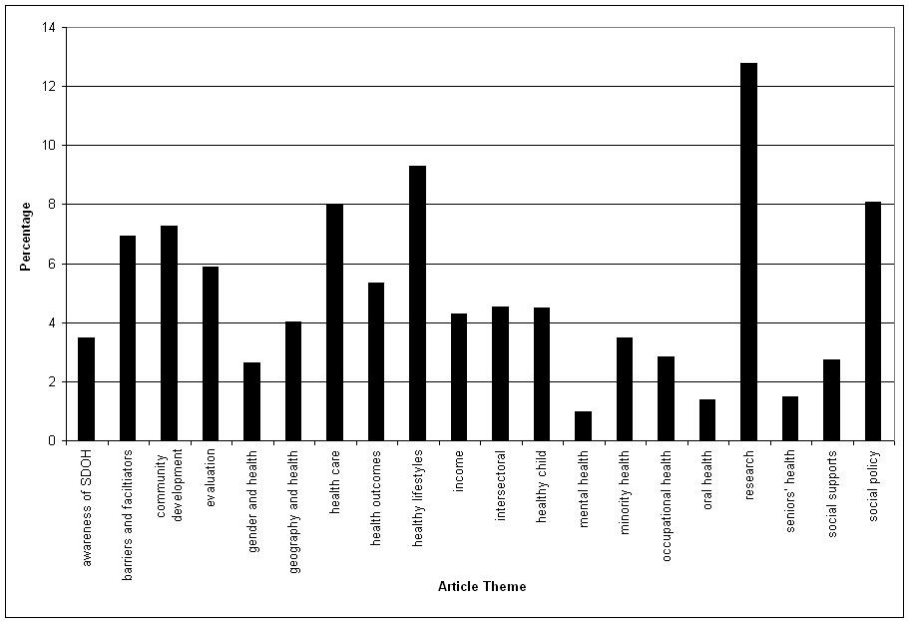
**Distribution of Article Themes as Percentage of Total Sample (*n *= 1004)**.

The distribution of article themes is displayed in Figure 2. The four most prominent themes are research (12.8%), healthy lifestyles (9.3%), social policy (8.1%), and healthcare (8.0%). Abstracts discussing research-related themes tended to focus on gaps in the knowledge base, conceptual issues and debates related to health inequities, developing and employing indicators/instruments/methods for assessing the scope of inequities or impacts of intervention, and challenges to knowledge translation. Abstracts covering healthy lifestyles discussed issues ranging from diet, to physical activity, substance use, and preventive screening. Social policy-themed abstracts discussed (the need for) upstream interventions, and described existing or potential social, public, health, or urban policies or plans. Topics in abstracts with healthcare themes ranged from health human resources, to service access and utilization, and primary care.

Health Canada's list of SDOH was used as the framework for identifying determinants mentioned in the abstracts [92]. Every abstract was assigned up to three SDOH, and abstracts that mentioned more than three SDOH were assigned a 'more than 3 SDOH' code. The six most commonly profiled SDOH were personal health practices and coping skills (*n *= 393), healthcare services and systems (*n *= 281), personal support networks and social inclusion (*n *= 239), social environments and social safety nets (*n *= 226), income and social status (*n *= 213), and physical and built environment (*n *= 192). (These tallies do not include abstracts that mentioned more than 3 SDOH because these abstracts tended to discuss health determinants in all-encompassing language and offered little detail about any specific determinants of health.) Thus, a substantial proportion of the health inequities knowledge base present lifestyle- and healthcare- (referred to in this article as 'behavioural' and 'biomedical', respectively) oriented perspectives regarding solutions to health inequities. Meanwhile, the high number of abstracts with social and physical environment SDOH profiles likely reflects the fact that the 'local' or 'municipal' level was one of four overarching search themes employed in the search strategy.

### Changes in Literature over Time

**Figure 3:**
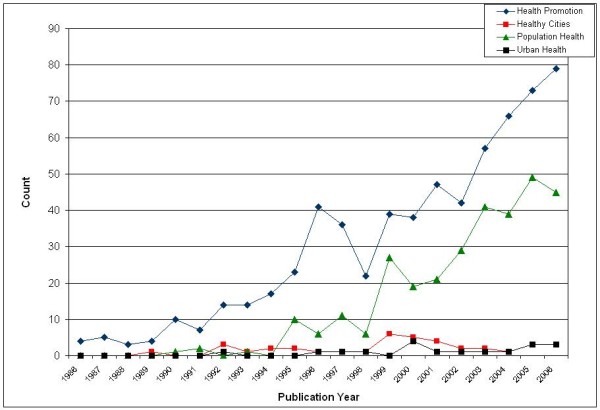
**Total Publication Activity over Time by Literature Body**.

The changes in publication activity in the four bodies of literature are displayed in Figure 3. Publication activity increased over the 20-year period, and the overwhelming majority of publications were generated from the HP and PH literature bodies. Publication activity in the HP and PH literatures increased almost every year, while the HC and UH literatures demonstrated slight increases in the 1999-2001 time period. These findings illustrate the growth of research programs on health inequities, and establishment of the health inequities knowledge base in the academy.

**Figure 4:**
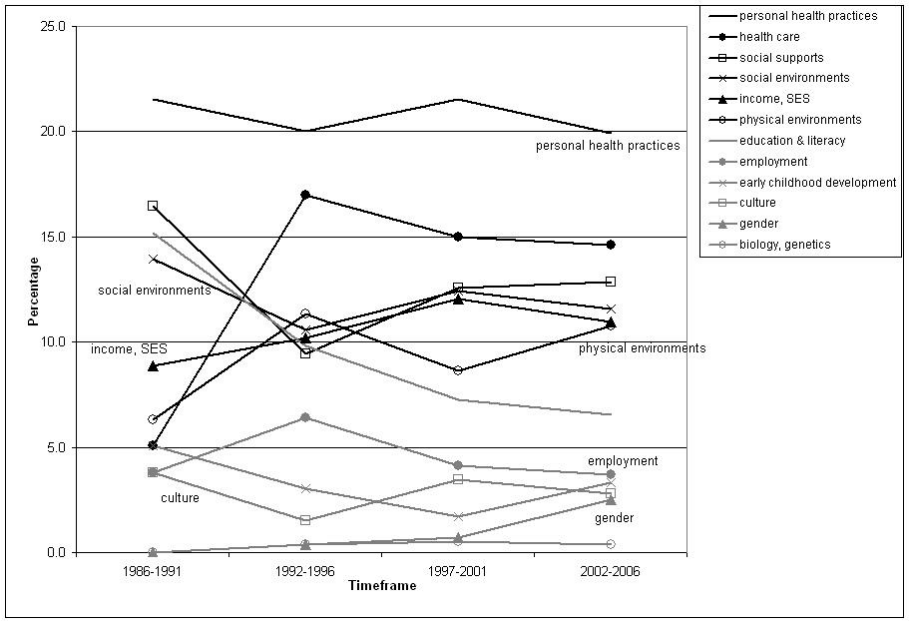
**Distribution of SDOH as Percentage by Five-Year Increments (*n *= 1918)**.

Changes in the SDOH profile of the article abstracts are displayed in Figure 4, using five-year increments to simplify the analyses. The 'personal health practices' determinant maintained the highest coverage over the entire time period, and discussions of 'healthcare' increased dramatically over time. Meanwhile, the determinants of 'education and literacy', 'social support networks' and 'social environments' started out relatively high, but lost their prominence over the remaining three timeframes. Taken together, these findings suggest that broader, more critical perspectives on health inequities were prominent in the early stages of development of the knowledge base, but that over time these perspectives gave way to a focus on 'behavioural' and 'biomedical' explanations for, and solutions to, health inequities.

### Implicating Municipal Governments

In the task of addressing health issues, 171 abstracts (17.0%) implicated municipal governments. The majority of HC (78.9%) and UH (55.6%) abstracts implicated municipal governments, while such implications were made in only a minority of abstracts from HP (14.7%) and PH (12.1%). Reflecting the rather "local" orientation of these fields, these findings suggest that the HC and UH literatures can more readily offer policy recommendations to municipal governments on interventions to reduce health inequities, while similar recommendations from the HP and PH literatures tend to be targeted at higher levels of government instead.

The geographic origins of these abstracts reveal some interesting trends. The majority of abstracts of a Mexican, Central and/or South American origin implicated municipalities (65%), while abstracts of American origin were least likely to implicate municipalities (8.4%). The greatest number of abstracts implicating municipalities emerged from Canada (*n *= 48) (likely owing to the sampling process that prioritized retrieving abstracts with a Canadian focus), while the fewest came from Asia, Africa & the Middle East (*n *= 11). The relatively large number (*n *= 41), and high percentage (33.9%), of abstracts implicating municipalities in the European literature suggests greater attention to the potential roles and responsibilities of municipal governments in addressing local health issues in this region.

**Figure 5:**
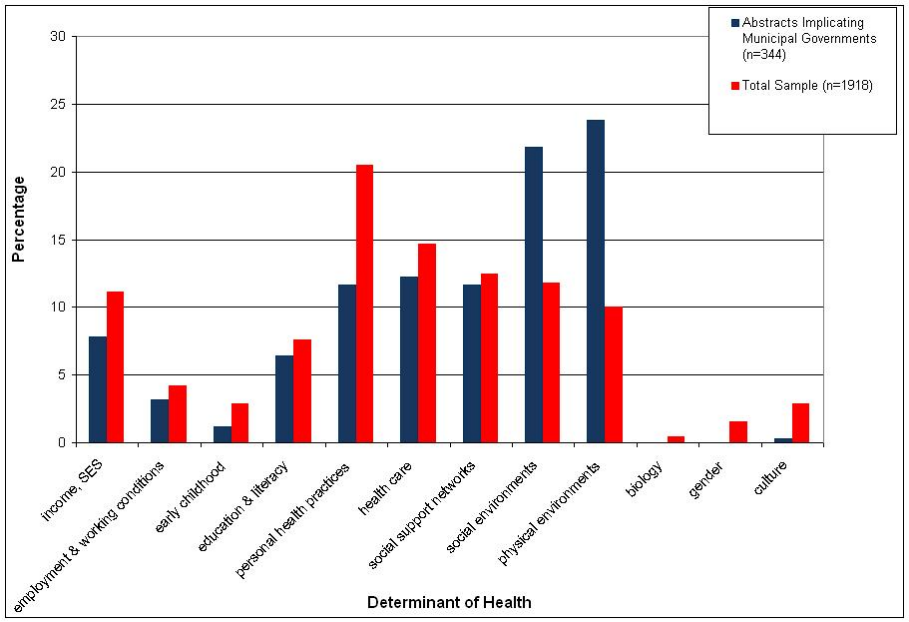
**Comparison of Combined SDOH Profile between Abstracts Implicating Municipalities versus Entire Sample**.

Comparisons made between the contents of the abstracts that implicated municipal governments (*n *= 171) and the entire sample (*n *= 1004) are depicted in Figure 5. Abstracts implicating municipalities focused more on local issues and environmental determinants of health, as compared to the sample as a whole that dealt more with 'biomedical' and 'behavioural' issues and determinants. The types of roles that were implicated, and the geographic origins of the abstracts that made those implications, are summarized in Table 2. As discussed in the methods, seven major categories of roles emerged from the literature review, through a thematic synthesis of the prescriptions made in all 171 abstracts. 'Joining or building on existing local health networks' (*n *= 41) and 'improving the social, economic, and built environment' (*n *= 39) were the two most commonly prescribed roles for municipal governments in the literature, while 'improving inter-governmental relations' was the least prescribed role (*n *= 12).

**Table 2:** Types of Roles Implicated and Geographic Origin of Abstracts Making Implications

	Global or trans-continental	Canada	Europe	Australia, New Zealand, Oceania	Asia, Africa & Middle East	Mexico, Central & South America	United States	Total Abstracts by Municipal Role
Roles	*n* (%)	*n* (%)	*n* (%)	*n* (%)	*n* (%)	*n* (%)	*n* (%)	*n* (%)
1. conduct health impact assessments, assess local needs	2 (6.9)	5 (10.4)	3 (7.3)	2 (16.7)	2 (18.2)	1 (7.7)	5 (29.4)	20 (11.7)

2. deliver health promotion, public education campaigns	0 (0.0)	4 (8.3)	5 (12.2)	2 (16.7)	3 (27.3)	3 (23.1)	2 (11.8)	19 (11.1)

3. develop inter-sectoral, intergovernmental partnerships	6 (20.7)	3 (6.3)	4 (9.8)	2 (16.7)	0 (0.0)	0 (0.0)	2 (11.8)	17 (9.9)

4. improve intergov'tal relations, clarify responsibilities	0 (0.0)	3 (6.3)	6 (14.6)	2 (16.7)	0 (0.0)	1 (7.7)	0 (0.0)	12 (7.0)

5. improve capacity w/n local government, be a leader, advocate	0 (0.0)	10 (20.8)	6 (14.6)	0 (0.0)	3 (27.3)	2 (15.4)	2 (11.8)	23 (13.5)

6. join/build on existing networks, partnerships, be an active participant	7 (24.1)	16 (33.3)	11 (26.8)	3 (25.0)	0 (0.0)	2 (15.4)	2 (11.8)	41 (24.0)

7. improve social, economic, built environments	14 (48.3)	7 (14.6)	6 (14.6)	1 (8.3)	3 (27.3)	4 (30.8)	4 (23.5)	39 (22.8)

Total Abstracts Implicating Municipal Governments	29 (100.0)	48 (100.0)	41 (100.0)	12 (100.0)	11 (100.0)	13 (100.0)	17 (100.0)	171 (100.0)

The seven categories of roles were emphasized to varying extents across the different geographical regions of origin. In abstracts of Canadian, European, and Australian & New Zealand origin, the most commonly prescribed role was to 'join or build on existing local health networks'. Canadian abstracts also emphasized the need for greater 'intra-municipal capacity building' to tackle local health issues. 'Improving the social, economic, and built environments' was the most commonly prescribed role among abstracts of a global/transcontinental origin, and of a Mexican, South & Central American origin, while abstracts of American origin stressed the need for municipalities to 'conduct health impacts assessments, and assess local needs'. The varying emphases placed on potential roles likely speak to the diverse jurisdictional responsibilities of municipal governments across and within countries, as well as the unique and highly specific health and social issues facing municipal governments within these countries. Accordingly, these differences signal the need for researchers to interpret these findings with caution by considering the applicability of these 'roles' within the context of a given municipal government's jurisdictional powers, functions, and public policy priorities.

Different themes were emphasized for each of the seven categories of municipal governments' roles. Abstracts that implicated municipal governments in 'conducting health impact assessments, assessing local needs' (role 1) stressed, for instance, the importance of collecting population-wide data on health and social needs at the municipal level [93]; utilizing data already available within municipal governments in planning local health and social services [94]; and engaging with local residents in identifying, and conceptualizing solutions to, local health problems [95].

Diverse prescriptions emerged for how municipal governments could become involved in 'delivering health promotion and public education programs on healthy lifestyles' (role 2). In the Canadian context, many of these prescriptions arose from the community-based heart health initiatives being implemented across the country [96]. Tobacco-cessation programs targeting children in disadvantaged neighbourhoods, for instance, clearly require the cooperation of municipal governments for their successful implementation [97]. In Tokyo, Japan, municipal Mayors are designating individuals to lead their communities to healthier lifestyles [98], while nearly a decade earlier, Rennes France incorporated health goals into all of its municipal decision-making [22].

Prescriptions for 'developing inter-sectoral, intergovernmental partnerships' (role 3) were broadest in scope and most similar across abstracts. These prescriptions generally emphasized the need for municipalities to form strong, functional relationships with senior levels of government to ensure that local governments have sufficient political and economic support to adequately address health and social issues at the local level [21,96,99].

For abstracts that prescribed the role 'improve intergovernmental relations, clarify jurisdictional responsibilities' (role 4), common themes stressed the importance of municipalities requiring a clear policy vision [100] and strategic direction [101] from senior governments to warrant prioritizing health inequities within municipal decision-making, as well as the necessary autonomy and authority to effectively address these issues at the local level [102].

Abstracts that prescribed 'improve capacity within local government' (role 5) for municipal governments tended to be broad in scope and similar across abstracts. The need for municipal governments to be strong leaders and advocates for addressing local health inequities were recurrent themes [103,104], as was the prescription that municipal policy-makers and program coordinators for health-focused initiatives have sufficient knowledge and expertise (i.e., capacity) to effectively lead such initiatives [105].

The commonly prescribed role of 'joining/building on existing networks and partnerships, being an active participant' (role 6) was diversely conceptualized across abstracts, in terms of relevant actors and types of networks that were emphasized. Some abstracts spoke generally about the need for institutionalized local public health networks with municipal governments as key contributors [106], while others offered more specific discussions of how a range of actors (including municipalities) could facilitate a model of health promotion at the local level [107]. Canadian abstracts discussed the results of municipalities working with local health units and community agencies on the SDOH [108], as well as the opportunities presented by the school setting to gather diverse actors to implement health promotion programs locally [109].

Abstracts that prescribed 'improving social, economic, built environments through public policy' emphasized the need for municipal governments to improve the social conditions of daily living in cities. Some abstracts adopted broad perspectives, discussing the need to reconnect public health and urban planning [74], while others focused on specific issues such as the provision of low-income housing [110], or the links between socio-spatial inequities and elementary school performance [111]. Abstracts of Mexican, South & Central American origin tended to stress the need for municipal governments to develop basic infrastructure and services (i.e., sewage, water filtration, waste removal) to facilitate healthier living conditions [112], signalling the stark contrast in health issues, and responsibilities therein, that confront municipalities in the developing world.

## Discussion

### Percentages, Timing, and Characteristics of Abstracts Reviewed

A total of 1004 abstracts were reviewed for the meta-narrative mapping exercise, with 94% of the abstracts emerging from the HP (*n *= 641) and PH (*n *= 307) literatures combined. That the HC and UH abstracts would constitute only 6% of the overall sample of abstracts is not surprising for several reasons. Rather than an academic line of inquiry per se, HC is a worldwide health movement designed to empower communities and cities to take action on locally defined health concerns [18]. The movement speaks to, and receives broad-based support from, governmental and non-governmental organizations alike, and consequently focuses its dialogue in the 'grey' literature that is not captured by academic databases [24,25,84,113]. Meanwhile, the total number of UH abstract hits was smaller than the other bodies of literature, as UH did not emerge as a distinct field of research until the early 2000s [42,114]. This small pool of abstracts, coupled with the fact that they most often did not fit the inclusion criteria (28% inclusion rate), generated a very small proportion of UH abstracts to be included in the meta-narrative mapping.

Publication activity in all four bodies of literature increased over time. The HP and HC abstracts dominated the first decade of the review, and the PH and UH literatures became more prolific over the second decade, mirroring the timelines of key developments in each of these fields of research. The publications of the Ottawa Charter and Epp Report in 1986 coincided with the emergence of HP [15,90] and the birth of the HC movement in Canada and internationally [17,115], while PH gained considerable momentum in the mid- to late-1990s [34,116,117], and UH emerged in the early 2000s [114,118].

Abstracts of Canadian origin were especially high among the PH literature, likely reflecting the strong influence of Canadian scholars in the development of this discourse [34,41,119,120]. Abstracts of European origin were most common among the HC literature, reflecting the fact that while the HC movement originated in Canada [115], Europe, facilitated by its support from the WHO regional office [121], has been at the forefront of HC policy interventions [21,122,123]. The greatest concentration of abstracts originating from developing countries were from the UH literature, as much of the current UH research focuses on detrimental health impacts of rapid urbanization [124-126]. Similar findings on the geographic origins of the health inequities knowledge base, especially of articles emerging from the HP and PH bodies of literature, have been observed elsewhere [12].

The epistemological traditions of the four bodies of literature likely account for the trends in study types and target populations that were observed. With their strong epidemiological roots [127,128], population-based surveys are commonly employed in the PH and UH literatures, and accounted for the majority of study types in this review [129]. Meanwhile, the orientation of the HC movement to community- and government-based action accounts for the preponderance of program evaluations. Finally, the relative diversity of study types and target populations among the HP literature likely reflects the age, maturity, and resulting diversity of research programs within this body of literature. It is also worth noting that the substantive scopes and methodological paradigms employed in studies from all four of these bodies would have been shaped, if not dictated, by the priorities and terms of funding agencies and requests for proposals.

### Thematic Contents of Literature and Changes over Time

Four article themes were particularly prominent in the abstracts reviewed. 'Research-related' themes, constituting 13% of article themes, captured issues ranging from conceptual or theoretical concerns (e.g., debates between PH and HP), appropriate use of indicators, instruments, and methods (e.g., how best to measure income inequality), and assessments of knowledge gaps and translation (e.g., lack of program evaluations). The highest proportion of research-themed articles occurred in the first quarter, with a steady decline in the remaining 15 years of the review. That research themes were the most prominent, especially early on in the review timeframe, suggests early efforts to establish a coherent body of knowledge on health inequities, and ongoing challenges in this knowledge base to developing evidence-based policy.

The other three themes that occurred in roughly equal measure (≈8% each) were 'healthy lifestyles' (i.e., consumption of alcohol and tobacco, nutrition and physical activity, preventive screening, and vaccines), 'healthcare' (i.e., access and utilization, costs and expenditures, systems, delivery, primary care, and health human resources), and 'social policy' (i.e., social, public, health, urban planning or policy). The prominence of the 'healthy lifestyles' and 'healthcare' themes illustrate the ongoing tendencies - criticized decades earlier [10] - for researchers to fixate on issues and interventions of a 'behavioural' and 'biomedical' nature. The prominence of the 'social policy' article theme might have suggested that a broader academic dialogue on health inequities was taking place. However, timeframe analysis revealed that 'social policy' coverage waned over the 20 year period timeframe, while coverage increased and remained consistently high over the 20 years for 'healthcare' and 'healthy lifestyles', respectively.

Similar findings were observed for the SDOH profile of the literature. The three most commonly profiled determinants - personal health practices & coping skills, healthcare services, and social support networks - reinforce the individualistic perspectives on population health inequities that emerged in the article theme analysis. While broader determinants, such as 'social environments', 'income & social status', and 'physical environments', were profiled, they constituted only 10% to 15% of all SDOH coverage over the entire 20 year time period. In contrast, coverage of 'personal health practices & coping skills' was at or above 20% over the entire timeframe, and 'healthcare' coverage increased considerably over time (from 5% in the first quarter to nearly 15% in the last). Thus, while some health inequities scholars made consistent attempts to steer the discourse towards broader health determinants and related implications that may be politically unpalatable, it appears there was a greater propensity to fixate on health determinants with implications for more downstream interventions that are often more amenable to implementation.

### Roles for Municipal Governments

Less than one-fifth (17%) of the abstracts implicated municipal governments in any way. The apparent inattention of the majority of health inequities researchers to municipal governments may be explained by a few reasons: they may simply not hold interests in this particular realm; they may struggle to access funding for research on municipalities; or they may recognize the limitations of municipal governments' capacities to address health inequities and consequently refrain from invoking municipalities' participation and/or target their recommendations to higher authorities. We are unable to discern from our study findings the extent to which any of these, or other factors, contribute to this observation.

Seven categories were established for potential municipal roles, responsibilities and activities to reduce population health inequities (Table 2). In the Canadian context, categories 1 and 2 deal with assessing health and social needs and delivering health-based services - assessments and service delivery that might typically fall outside the range and jurisdiction of municipal services [95,130]. Categories 3 through 6 deal with relationships between the municipality and other governments, non-governmental organizations, and within the municipality itself [84], while category 7 captures the types of responsibilities over which Canadian municipalities have clear existing jurisdiction, such as zoning, by-law enforcement, public libraries, and fire protection [68].

While abstracts of Canadian origin implicated municipalities the most (*n *= 48), the proportion of these relative to all Canadian abstracts reviewed was relatively small (11%). This finding suggests that the overall Canadian contribution to the health inequities knowledge base has been minimal in terms of prescriptions for municipal activity on health inequities. In contrast, while small in number (*n *= 13), the majority of abstracts of Mexican, South & Central American origins (65%) implicated roles for municipalities. The municipal level focus in this region of the world is likely attributable to a few factors: 1) the 'local' nature of many health problems, whereby cities in Mexico, South & Central America are simultaneously lacking basic municipal infrastructure and services to facilitate sanitary living conditions [131-133], *and *face common Western-world health problems associated with rapid urbanization (e.g., pollution-induced asthma) and widespread adoption of sedentary lifestyles (e.g., obesity) [132,134]; 2) the influence of the Pan American Health Organization (PAHO) which has played a key role in addressing population health inequities in Latin America, including conducting research on strategies for engaging municipalities in health promotion initiatives [135] and providing strategic direction for developing interventions to address 'neglected populations' [136]; and 3) higher investments in participatory community-based approaches to tackling local health and social issues, as well as a strong tradition of engagement with the Healthy Cities movement in these countries [137-141].

Considering both the total number (*n *= 41) and the proportion (33%) of abstracts implicating municipalities, it would appear that the European literature has made the most substantial contribution to the academic dialogue on prescriptions for municipal governments to address local health inequities. The emphasis placed on municipal governments by the European abstracts mirrors the importance placed on healthy urban planning and the prominence of the Healthy Cities movement in the European context [85,123]. With comparable (if not superior) municipal infrastructures and population health profiles, prescriptions arising from European literature bear some relevance and utility to the North American context. Indeed, it is worth noting that 'joining or building on existing local health networks and partnerships' was the most commonly cited role in both the European and Canadian literatures, suggesting that similar challenges and contexts for municipal intervention exist in these distinct geographical regions.

### Limitations

The most important limitation of our study is in attempting to make generalizations about the applicability of potential municipal government interventions across diverse governmental forms and functions, and geographical jurisdictions. As discussed in the introduction, the scope of powers and responsibilities of municipal governments vary tremendously both across and within nations. The generalizability of the study findings was enhanced through the use of more generic terms to code the abstracts, and by synthesizing the full scope of the scholarly 'prescriptions' into seven broadly defined and internationally relevant categories; by employing this thematically broad codebook for extracting data from the abstracts, researchers and policy-makers are permitted greater latitude to conceptualize municipal interventions relevant to their own jurisdictions. Despite a rigorous methodological design, the nature of any meta-analysis requires readers need to be critical in applying study findings to the unique contexts in which they work.

While the literature search was international in scope, the priority placed on abstracts of Canadian origin and the exclusion of non-English language abstracts mars our findings with a 'Western hemisphere' or 'developed country' bias. The English language is predominant in primarily wealthy nations whose researchers have disproportionate access to research funding and success with publication; have well established municipal governance systems and sophisticated municipal infrastructure; and have high functioning acute-care medical systems, and public health sectors that deal increasingly with reducing chronic, rather than infectious, diseases. Meanwhile, there is tremendous international variation in the scope of, and patterns in, population health inequities, and no internationally agreed upon definition for 'population health inequities'. These characteristics have important implications for the nature of municipal governments' involvements in addressing population health inequities, and thus, likely influenced the scope of prescribed roles that emerged from the literature reviewed for this study. Had our language capacities facilitated it, this limitation could have been partly addressed by reviewing articles of non-English origin. However, we submit that this 'Western hemisphere' bias is not isolated to our study, but rather pervades academe in general, and is especially reflected in developing country researchers' inequitable access to research funding and publication acceptation in international journals. It is possible that other prescriptions for municipal-level involvement (likely focusing on developing basic infrastructure and provision of relief aid in partnership with non-governmental organizations) may have emerged if more abstracts been reviewed from researchers in developing countries. Given the implicit interests in this study in understanding potential roles for municipalities with established and operational governance structures, we feel that the breadth of data retrieved from the abstracts that *were *reviewed remains applicable and relevant to jurisdictions that may have been under-represented in our analysis.

Another limitation of this study was in restricting our analysis to the four bodies of literature chosen. As discussed, our decision not to include the policy sciences and social epidemiology, for instance, may have led our findings to under-represent dimensions of the health inequities knowledge base that focus on broader social welfare policies or more technically-oriented epidemiological studies documenting the scope of health inequities at the local level. As we were interested in uncovering scholarly prescriptions for municipal government *interventions *on *health *inequities, we feel that the breadth of the search strategy that was employed (in terms of scope of electronic databases and search terms) captured the abstracts of greatest relevance to the questions posed in this study.

A related limitation was in treating these four bodies of literature as discrete and mutually exclusive entities. These bodies of literature co-developed over the past two decades, and as with most academic disciplines with diverse perspectives, they rely on the same baseline information. Indeed, 51 of the abstracts reviewed appeared in more than one body of literature and accounted for a total of 103 abstract cases in the dataset. An analysis of the differences between the contents of the repeat abstracts and the total sample was performed (results not shown), to document any systematic differences in articles that permeate multiple literatures. While there were no significant differences in geographic origin or in the relative emphasis on categories for municipal roles, compared to the entire sample, these 51 abstracts were significantly more likely to focus on the '*social environment*' determinant of health (p = 0.002) and to implicate municipal governments in the task of addressing health inequities (p < 0.001). Thus, while this approach was employed to ensure methodological transparency and the accurate depiction of the relative contribution of each body of literature, this sub-analysis reveals that the actual quantity of abstracts emphasizing broader health determinants and a role for municipal governments was slightly overestimated and that, in fact, health inequities scholars have been even less vocal on these issues than what our larger analysis suggests.

Having a second reviewer would have been beneficial for confirming the validity and reliability of the codebook, but this was not possible due to inadequate study funding. Similarly, not reviewing entire articles may have presented an analytical weakness in this study, as article abstracts typically provide only cursory information; the information requirements for abstracts vary considerably across journals; and relevant articles without abstracts would have been excluded. Reviewing entire articles would have revealed a more accurate picture of the nuances of the health inequities knowledge base, but the sample size would have necessarily been smaller to facilitate such an intense review. Because of the importance of abstracts in offering readers a "preview of what's to come" while emphasizing some issues over others, the more cursory approach of reviewing abstracts was the best way to track meta-narratives from this large and diverse body of knowledge over a twenty year timeframe.

### Policy Implications: Prescriptions for Municipal Government Intervention

Overall, the health inequities knowledge base offered insufficient guidance to municipal governments in developing healthy public policy at the local level. Health was conceptualized in primarily 'behavioural' and 'biomedical' terms, providing little incentive for municipalities to consider, and act on, the full range of the SDOHs. If researchers, who have at their disposal voluminous evidence on the social determinants of health inequities, overwhelmingly defer to healthy lifestyles and healthcare services as the levers for improving health, then how can busy, and often uninformed, policy-makers be expected to conceptualize health any differently? The minimal attention paid to municipal governments in the health inequities knowledge base urges critical reflection on the subject areas and types of health research that funding agencies privilege, and highlights the need for increased funding and translation of interdisciplinary health inequities research that is relevant to policy-makers, especially at the municipal level where human resources devoted to exchange with research communities are in short supply.

The relative silence of the health inequities knowledge base on avenues for municipal action presents another challenge to developing healthy public policy at the municipal level. With less than one-fifth of the abstracts implicating municipalities in any way, and the tendency for those implications to originate from Europe, it is clear that health inequities researchers offer inadequate prescriptions for municipal policy-makers from other jurisdictions to draw from [142]. Even if prescriptions were readily available, municipal policy-makers would justifiably have little faith in the effectiveness of such prescriptions, given the dearth of evaluations of programs targeting health inequities [143]. At a minimum, though, paradigm shifts are needed in both the academic and policy domains to move the issue of population health inequities onto the municipal government agenda.

### Future Research Directions

The findings from this study illuminate a number of potential avenues for future research. Given its explicit 'city' focus, it was not surprising that the Healthy Cities literature implicated municipal governments in the greatest proportion. One might have expected, however, to see more implications from the Canadian Healthy Communities literature, considering the role that Canadian scholars played in launching the Healthy Cities movement. What characteristics are present in Europe, that are not present in North America and other jurisdictions, that would explain the apparent uptake of Healthy Cities agendas by municipal policy-makers across the region (as evidenced by the various past and current Healthy Cities projects across Europe), as well as the high degree of support from the WHO regional office for Healthy Cities programs? What powers might European cities possess that facilitate the implementation of prescriptions from the Healthy Cities movement? These cross-jurisdictional differences signal the need for researchers to investigate the nature of urban health governance across diverse political systems, which could offer an explanation for the lack of action on health inequities at the local level in Canada and elsewhere.

While our findings suggest evidence of uptake of the Healthy Cities component of the health inequities knowledge base by municipal policy-makers, especially within European and Latin American jurisdictions, little is known of the extent of uptake of the other components of this knowledge base by municipal policy-makers. As the objective of this study was to survey the health inequities knowledge base for prescriptions for municipal government intervention, another logical direction for future research would be to assess the extent of awareness and utilization of this knowledge by municipal policy-makers in jurisdictions around the world, as well as the perceptions held by municipal policy-makers themselves of the roles and responsibilities of municipalities in addressing health inequities. In another component of this study, we *have *investigated these questions within the metropolitan region of Metro Vancouver, Canada, which consists of nearly twenty autonomous municipal governments [144]. Similar analyses are needed in other jurisdictions.

In addition to investigating the status of translation of the health inequities knowledge base by municipal policy-makers, regular efforts should also be made to review the knowledge base for emergent prescriptions for both governmental and non-governmental interventions. In August 2008, the WHO Commission released another report on the state of the SDOH and strategies for reducing population health inequities [145]. The dynamic and evolving nature of this knowledge base suggests that the relevance of, and academic support for, strategies and interventions to reduce population health inequities can be short-lived.

## Conclusions

It is well established that municipal governments have a fundamental influence on creating, and potentially reducing, health inequities in cities. The early links in the 19th century between urban planners and public health practitioners facilitated dramatic improvements in the living conditions of city dwellers in the developed world, and set the stage for considerable improvements in longevity over the next century. And, after decades of silence, the importance of our daily living conditions - conditions that are so fundamentally shaped by municipal government policies - has re-emerged within the field of public health as a key determinant of the health of populations. Yet, despite the discursive shift in public health, and the establishment of several academic disciplines examining health inequities in varying capacities since this shift, the precise roles and responsibilities of municipal governments in reducing health inequities at the local level have been inadequately investigated and remain poorly understood.

This study summarizes scholarly prescriptions for municipal government interventions on local health inequities. These prescriptions included partnerships with other levels of government or locally-based non-governmental organizations, assessments of local needs, delivery of health promotion and education programs, enhancing capacities within municipal governments, and investing in existing municipal infrastructure and programming. While biomedical and behavioural perspectives were pervasive in the knowledge base on the whole, abstracts that implicated municipalities tended to employ 'structure-oriented' perspectives that dealt with broader social policy issues. Despite the contents and orientations of these prescriptions however, municipalities received limited overall attention from the scholarly domain.

It may be unreasonable to expect municipalities to glean useful insights from the relatively sparse prescriptions that have been made, or that these limited prescriptions bear relevance to their jurisdictional contexts and health concerns. An understanding of the capacities for urban health governance in diverse political systems is required among readers to critically assess these expectations. To truly reinvigorate the link between public health and urban planning, more research is needed on the extent of knowledge translation of the health inequities knowledge base at the municipal level, as well as the willingness and capacities of municipal governments to intervene on these issues. The findings presented here, which offer unique insights into how municipalities have been implicated by the research community, are a critical step in the journey of translating knowledge into government interventions to reduce population health inequities.

## Competing interests

The authors declare that they have no competing interests.

## Authors' contributions

PC conceived of, and implemented the study design; retrieved and analyzed all of the article abstracts; and developed a draft of the manuscript. MH assisted with the conception of the study and the development of the abstract codebook and search strategy; and assisted in the writing of the manuscript. All authors read and approved the final manuscript.

## Supplementary Material

Additional file 1 **Article Abstract Codebook**. The table provided summarizes all of the article variables and corresponding codes that were employed to extract data from the *n *= 1004 article abstracts. Click here for file
