# Supplementary material for: The role of urban municipal governments in reducing health inequities: A meta-narrative mapping analysis
Source: Int J Equity Health. 2010 May 25;9:13. doi: 10.1186/1475-9276-9-13 (PMC2893183; doi:10.1186/1475-9276-9-13)
Supplement: Additional file 1 — Article Abstract Codebook. The table provided summarizes all of the article variables and corresponding codes that were employed to extract data from the n = 1004 article abstracts. [file 1475-9276-9-13-S1.PDF]

Variable Name	Codes
**Bibliographic Variables**	
Body of Literature	HP, HC, PH, UH
Journal Name	(String variable)
Publication Date	Year
Geographical Origin	-Global, transcontinental<br>-Canada<br>-Europe<br>-Australia, New Zealand, Oceania<br>-Asia, Africa, Middle East<br>-Central, South America, Mexico<br>-United States
Study Type	-Population-Based Survey<br>-Experimental, Quasi-Experimental<br>-Program Evaluation/Description<br>-Case Study, Qualitative, Mixed-Methods<br>-Review (Systematic, Conceptual)<br>-Commentary
**Abstract Content Variables**	
Article themes	-Awareness of SDOH, attitudes towards addressing health inequities<br>-Barriers and facilitators to addressing SDOH, health inequities, improving health outcomes<br>-Community development, participation, capacity building, mobilization, social capital or cohesion, impacts or needs assessments<br>-Evaluation or description of health promotion, public health interventions<br>-Gender and health, sexual health<br>-Geography, place, location, environments and health<br>-Healthcare – access, utilization, costs, expenditures, systems, delivery, primary care, human resources<br>-Health outcomes - disabilities, diseases, illnesses, injuries, mortality<br>-Healthy lifestyles – general, diet, exercise, preventive screening, vaccines, alcohol, smoking, drugs, sun exposure, etc.<br>-Income, SES, social class, wealth and health, social gradients<br>-Inter-sectoral collaboration, coordination, comprehensive, inter-disciplinarity, partnerships

	-Healthy child development - maternal, prenatal, neonatal, children, youth, school health<br>-Mental health (including mental illness)<br>-Minority health (Aboriginals, immigrants, refugees, other minorities)<br>-Occupational, employee health and worksite interventions<br>-Oral health (oral hygiene, dental interventions)<br>-Research (conceptual, training, indicators, instruments, methods, knowledge translation, knowledge gaps)<br>-Seniors' health<br>-Social supports, social inclusion, self-efficacy, care-giving, self-empowerment<br>-Social, public, health, urban policy or planning, SDOH, upstream factors, broader context
SDOH	-Income and socioeconomic status<br>-Employment and working conditions<br>-Healthy child development<br>-Education and literacy<br>-Personal health practices & coping skills<br>-Health services<br>-Social support networks<br>-Social environments<br>-Physical environments<br>-Biology & genetic endowment<br>-Gender<br>-Culture<br>-More than 3<br>-None specified
**Municipal Prescription Variables**	
Implicates Municipal Government	Yes/No
Types of Municipal Roles Implicated	(String variable)
